# Supplementary material for: Capturing the emergent dynamical structure in biophysical neural models
Source: PLoS Comput Biol. 2025 May 12;21(5):e1012572. doi: 10.1371/journal.pcbi.1012572 (PMC12068601; doi:10.1371/journal.pcbi.1012572)
Supplement: S1 Appendix — Granger causality is explained in relation to transfer entropy, with details on least-squares prediction, statistical testing, and computational methods. Alternative estimation techniques and software implementation are also discussed. (PDF) [file pcbi.1012572.s001.pdf]

Supporting information

S1 Appendix: Granger causality

Estimating transfer entropy comes with some key challenges [1]. As a conditional mutual information, the state-of-the-art estimator uses the Kraskov–Stogbauer–Grassberger (KSG) nearest-neighbour algorithm [2–4], though kernel methods are also popular [3]. Due to inherent limitations of nearest-neighbour methods, the KSG estimator tends to overestimate TE, resulting in positively biased estimates (see Bossomaier *et al.* [1] for an in depth exposition). Furthermore, in lieu of known analytic sampling distributions, subsampling techniques must be employed for statistical testing, which may not systematically mitigate the bias inherent in nonzero TE estimates [5, 6]. Another issue with nonparametric estimators such as KSG, is that due to a curse of dimensionality which relates to the number of time-lags used in both source and target variables for TE estimation. In practice, one can restrict to just a few time lags, but this is known to risk skewing TE estimates [7]. Because of the inherent computational inefficiency and lack of a known analytic sampling distribution for statistical inference we leverage the close relationship between Transfer entropy and Granger causality [8, 9]. We remark that, unlike transfer entropy, Granger causality may be decomposed in the spectral domain; we don't however make use of spectral Granger causality in this study.

Transfer entropy and Granger causality are equivalent under the assumption that the stochastic processes involved are jointly Gaussian [8][1]; the equivalence extends to a broader class of exponential distributions [10]. More generally, for arbitrary (possibly nonlinear) Markovian predictive models, if the GC statistic is construed as a log-likelihood ratio (see below), then GC is asymptotically equivalent to TE in the large-sample limit [9]. Granger causality may be viewed, then, as an approximation to transfer entropy, and inherits the interpretation of the latter as *information flow.* In comparison to transfer entropy, Granger causality is generally more efficient to estimate, is able to accommodate longer histories, and common estimators have smaller bias and variance, as well as known analytic sampling distributions.

Granger causality reframes transfer entropy in terms of least-squares optimal prediction, as opposed to (conditional) mutual information. Suppose given a stationary, multivariate, continuous-valued, zero-mean stochastic process $\boldsymbol{U}$ (the *universe of information* [11]) specified by $\boldsymbol{U}_t = \left[\boldsymbol{X}_t^\top\, \boldsymbol{Y}_t^\top\, \boldsymbol{Z}_t^\top\right]^\top$, which is partitioned into three sub-processes $\boldsymbol{X}$, $\boldsymbol{Y}$ and $\boldsymbol{Z}$. We say that the source variable $\boldsymbol{X}$ Granger-causes the target variable $\boldsymbol{Y}$ conditional on $\boldsymbol{Z}$ if the prediction of $\boldsymbol{Y}$ based on its own past and the past of $\boldsymbol{Z}$ is enhanced by the addition of the past of $\boldsymbol{X}$ to the predictor set, where prediction is in the least-squares sense, and (following [12]) quantified by the log-generalised variance[2] [13] of the residual prediction error[3].

More precisely, the *full* optimal least-squares prediction of $\boldsymbol{Y}_t$, based on the joint past $\boldsymbol{U}_{t-\tau}$ of all variables[4], is given by the conditional expectation

$$\mathbb{E}[\boldsymbol{Y}_t \,|\, \boldsymbol{U}_{t-\tau}]\,, \tag{1}$$

with residual error

$$\boldsymbol{\varepsilon}_{y,t} = \boldsymbol{Y}_t - \mathbb{E}[\boldsymbol{Y}_t \,|\, \boldsymbol{U}_{t-\tau}] \tag{2}$$

[1]More precisely, any finite subset of process variables at arbitrary time stamps has a multivariate-normal distribution.

[2]The generalised variance of a random vector is the determinant of its covariance matrix.

[3]Besides stationarity, the GC formalism requires some further regularity conditions on the process $\boldsymbol{U}$; for all stochastic processes in this study we assume the same conditions as described in [12, §2, p. 305].

[4]On the analytical level, prediction is based on the *infinite* past. For empirical data, histories will of necessity be truncated at some suitable number of lags.

(the y subscript indicates that the target (predicted) variable is $\boldsymbol{Y}$). We use the notation $t-\tau$, where τ is not a single value but represents a set of lagged time points, $\tau = 1, 2, 3, \ldots, n$, referring to the history of the process. In the case of an infinite history $\tau = 1, 2, 3, \ldots$, this set would extend indefinitely.

The *restricted* (or *reduced* prediction of $\boldsymbol{Y}_t$ is based on the joint past of the restricted process $\boldsymbol{U}^{\mathsf{R}}$ given by $\boldsymbol{U}_t^{\mathsf{R}} = \left[\boldsymbol{Y}_t^\top \, \boldsymbol{Z}_t^\top\right]^\top$, where the source variable $\boldsymbol{X}$ is omitted (we generally denote restricted quantities by a superscript "R"):

$$\mathbb{E}\left[\boldsymbol{Y}_t \,|\, \boldsymbol{U}_{t-\tau}^{\mathsf{R}}\right], \tag{3}$$

with residual error

$$\boldsymbol{\varepsilon}_{y,t}^{\mathsf{R}} = \boldsymbol{Y}_t - \mathbb{E}\left[\boldsymbol{Y}_t \,|\, \boldsymbol{U}_{t-\tau}^{\mathsf{R}}\right]. \tag{4}$$

The full and restricted prediction error covariance matrices are respectively

$$\Sigma_{yy} = \mathbb{E}\left[\boldsymbol{\varepsilon}_{y,t}\boldsymbol{\varepsilon}_{y,t}^\top\right] \quad \text{and} \quad \Sigma_{yy}^{\mathsf{R}} = \mathbb{E}\left[\boldsymbol{\varepsilon}_{y,t}^{\mathsf{R}}\boldsymbol{\varepsilon}_{y,t}^{\mathsf{R}\top}\right] \tag{5}$$

and, following [12], the Granger causality from $\boldsymbol{X}$ to $\boldsymbol{Y}$, quantifying the gain in prediction efficacy by inclusion of the source history $\boldsymbol{X}_{t-1\,:\,-\infty}$ in the predictor set, is then defined as the log-ratio of generalised variances

$$F(\boldsymbol{X} \to \boldsymbol{Y} \,|\, \boldsymbol{Z}) = \log \frac{|\Sigma_{yy}^{\mathsf{R}}|}{|\Sigma_{yy}|} \tag{6}$$

If there are no conditioning variables, then the above goes through with $\boldsymbol{Z}$ omitted.

The most common application of Granger causality to neurophysiological data is derivation of the *(Granger-)causal graph*. Given a multivariate (vector) process $\boldsymbol{X}$ with $\boldsymbol{X}_t = [X_t^1, \ldots, X_t^N]^\top$, where the univariate processes X^i commonly represent single channels or regions of recorded data associated with specific anatomical brain regions, this is the directed, weighted graph of pairwise-conditional Granger causalities, defined as

$$G_{ij}(\boldsymbol{X}) = F\Big(X^j \to X^i \,|\, \boldsymbol{X}^{[ij]}\Big), \tag{7}$$

where the $[ij]$ superscript indicates that the variables X^i and X^j are omitted from the full process $\boldsymbol{X}$. Thus $G_{ij}(\boldsymbol{X})$ represents the directed GC from channel j to channel i, controlling for indirect influences from other channels. The causal graph is often taken to summarise directed functional connectivity for neural systems.

In sample, the optimal predictions (conditional expectations) (1), (3) may be estimated from the data by standard Ordinary Least Squares (OLS), or another maximum-likelihood estimator, and if $\boldsymbol{U}$ is a Gaussian process, then the sample statistic corresponding to (6) is a log-likelihood ratio (*cf.* [9]); as such, under the null hypothesis of vanishing GC it has an asymptotic χ^2 distribution[5].

The above exposition suggests that in sample both full and restricted predictions must be estimated separately; in practice, this is equivalent to estimating (full and restricted) vector-autoregressive (VAR) models. There is also a *single-regression* VAR estimator, where the restricted residual error distribution is derived analytically from the full model estimate. This estimator has smaller bias and variance than the conventional dual-regression estimator, and the null sampling distribution is known, at least in the unconditional case, to be a generalised χ^2 [14]; see [15].

Alternative routes to estimation of Granger causality beyond VAR modelling exist: the first is a parametric approach based on linear state-space modelling, while the second, nonparametric method is based on *spectral factorisation* of the cross-power

[5]The null hypothesis of vanishing GC may also be tested by an F-test, which anecdotally is more powerful than the χ^2 test, especially for small samples

spectral density (CPSD) matrix of the full process $\boldsymbol{U}_t$. We discuss the latter below, and introduce state-space modelling in S2 Appendix. Efficient MATLAB implementations of all estimation methods described here are publicly available as open-source software in the Multivariate Granger Causality Toolbox, version 2 (MVGC2 [16, 17]), which may be obtained from https://github.com/lcbarnett/MVGC2.

Calculating Granger causality via spectral factorisation

The CPSD of a stationary, zero-mean process $\boldsymbol{U}$, written $S(\omega)$, is defined as the two-sided Fourier transform of the *autocovariance sequence* $\Gamma_k = \mathbb{E}[\boldsymbol{U}_t \boldsymbol{U}_{t-k}^\top]$, $-\infty < k < \infty$. There are a variety of standard techniques for estimating the CPSD from time-series data, including averaged periodogram, multi-taper, and wavelet methods, or it may be calculated from parametric models.

At any circular frequency $\omega \in [-\pi, \pi]$, $S(\omega)$ is a Hermitian matrix. Under our assumed regularity conditions, $S(\omega)$ may be uniquely factorised as [19]

$$S(\omega) = H(\omega)\Sigma H^*(\omega) \tag{8}$$

where $H(z)$, $z \in \mathbb{C}$ is the *transfer function* for the process[6], superscript '*' denotes matrix conjugate transpose, and Σ is the residual error covariance matrix associated with the optimal least-squares prediction $\mathbb{E}[\boldsymbol{U}_t \,|\, \boldsymbol{U}_{t-\tau}]$ of $\boldsymbol{U}$ on its past. Given a CPSD at some frequency resolution, there are stable and efficient algorithms for effecting the *spectral factorisation* (8) [17, 19].

We exploit spectral factorisation to calculate the Granger causality $F(\boldsymbol{X} \to \boldsymbol{Y} \,|\, \boldsymbol{Z})$ for a partitioned process $\boldsymbol{U}$, $\boldsymbol{U}_t = \left[\boldsymbol{X}_t^\top \, \boldsymbol{Y}_t^\top \, \boldsymbol{Z}_t^\top\right]^\top$ as follows [20]: given the CPSD $S(\omega)$ for the full process $\boldsymbol{U}$, we obtain the residuals covariance matrix Σ_{yy} of (6) by factorising $S(\omega)$ according to (8) and taking the yy block of the factored residuals covariance matrix Σ. Next, we note that the yz block of $S(\omega)$ is just the CPSD of the restricted process $\boldsymbol{U}^{\mathsf{R}} = \left[\boldsymbol{Y}_t^\top \, \boldsymbol{Z}_t^\top\right]^\top$; again according to (8), we factorise this restricted CPSD $S^{\mathsf{R}}(\omega)$ to obtain a restricted residuals covariance matrix Σ^{R}, and take the yy block to obtain the Σ_{yy}^{R} of (6).

We also state here a useful classical result ([21]): under our assumed regularity conditions:

$$\frac{1}{2\pi}\int_{-\pi}^{\pi} \log|S(\omega)|\, d\omega = \log|\Sigma| \tag{9}$$

This equation connects the GC estimate obtained through VAR modelling to the spectral factorisation of the CPSD.

Dynamical dependence as a Granger causality

We consider the dynamical dependence $T(\boldsymbol{X} \to \boldsymbol{Y})$ for an N-dimensional microscopic process $\boldsymbol{X}$ on $\mathbb{R}^N$ and a coarse-grained n-dimensional macroscopic variable $\boldsymbol{Y}$ given by $\boldsymbol{Y}_t = M\boldsymbol{X}_t$, where M is a full-rank $n \times N$ matrix. As explained in the main text, without loss of generality we may assume that M is an orthogonal matrix: $MM^\top = I$. We examine the GC for the dynamical dependence, given by

$$F(\boldsymbol{X} \to M\boldsymbol{X}) \tag{10}$$

The formula (9) allows us to derive an elegant and computationally-efficient expression for the DD. Firstly, it follows straightforwardly that the restricted CPSD is given by

[6] By abuse of notation, we write $H(\omega)$ for $H(z)$ with $z = e^{-i\omega}$ on the unit circle in the complex plane.

$S^{\mathsf{R}}(\omega) = MS(\omega)M^\top$, so that we may calculate $\log\left|\Sigma^{\mathsf{R}}_{yy}\right|$ immediately from (9). Secondly, we have $\Sigma_{yy} = M\Sigma M^\top$, so that

$$F(\boldsymbol{X} \to M\boldsymbol{X}) = \frac{1}{2\pi}\int_{-\pi}^{\pi} \log\left|MS(\omega)M^\top\right| d\omega - \log\left|M\Sigma M^\top\right| . \tag{11}$$

Transformation-invariance of DD allows this to be simplified even further: we can always transform the microscopic space $\mathbb{R}^N$ in such a way as to decorrelate and normalise the residual prediction errors so that $\Sigma = I$, the identity matrix [18]. Then by orthogonality of M, the second term in (11) vanishes, and we obtain

$$F(\boldsymbol{X} \to M\boldsymbol{X}) = \frac{1}{2\pi}\int_{-\pi}^{\pi} \log\left|MS(\omega)M^\top\right| d\omega \tag{12}$$

Note that as to the utility of this formula, it does not matter *how* $S(\omega)$ and Σ have been acquired, e.g., whether it be via parametric modelling or spectral factorisation. It is particularly useful in a DD optimisation scenario: acquisition of $S(\omega)$ and Σ, followed by residuals decorrelation/normalisation, need only be performed once, and (12) may subsequently be applied as often as required for any set of M. The computational efficiency of (12) hinges on the dimensionality of N and n, and in particular the spectral resolution of $S(\omega)$, i.e., the size of the frequency increment $\Delta\omega$ in the numerical quadrature for the integral in (12); the largest acceptable frequency increment will be determined by the complexity of the spectrum of $\boldsymbol{X}$.

References

[1] Bossomaier T, Barnett L, Harré M, Lizier JT. An introduction to transfer entropy: Information flow in complex systems. An Introduction to Transfer Entropy: Information Flow in Complex Systems. 2016; p. 1–190. doi:10.1007/978-3-319-43222-9/COVER.

[2] Kraskov A. Synchronization and interdependence measures and their applications to the electroencephalogram of epilepsy patients and clustering of data. NIC-Secretariat, Research Centre Jülich; 2004.

[3] Lizier JT. JIDT: An information-theoretic toolkit for studying the dynamics of complex systems. Frontiers in Robotics and AI. 2014;1:11.

[4] Wollstadt P, Lizier JT, Vicente R, Finn C, Martinez-Zarzuela M, Mediano P, et al. IDTxl: The Information Dynamics Toolkit xl: a Python package for the efficient analysis of multivariate information dynamics in networks. arXiv preprint arXiv:180710459. 2018;.

[5] Vicente R, Wibral M, Lindner M, Pipa G. Transfer entropy—a model-free measure of effective connectivity for the neurosciences. Journal of computational neuroscience. 2011;30(1):45–67.

[6] Vicente R, Wibral M. Efficient estimation of information transfer. In: Directed Information Measures in Neuroscience. Springer; 2014. p. 37–58.

[7] Borisovsky P, Eremeev A. A Study on Performance of the (1+1)-Evolutionary Algorithm. undefined. 2002;.

[8] Barnett L, Barrett AB, Seth AK. Granger causality and transfer entropy Are equivalent for gaussian variables. Physical Review Letters. 2009;103(23). doi:10.1103/PHYSREVLETT.103.238701.

[9] Barnett L, Bossomaier T. Transfer Entropy as a Log-likelihood Ratio. Physical Review Letters. 2012;.

[10] Hlavackova-Schindler K, Hlaváčková-Schindler K. Equivalence of Granger Causality and Transfer Entropy: A Generalization Theory of Neural Networks View project Equivalence of Granger Causality and Transfer Entropy: A Generalization. Applied Mathematical Sciences. 2011;5(73):3637–3648.

[11] Granger CWJ. Investigating Causal Relations by Econometric Models and Cross-spectral Methods. Econometrica. 1969;37(3):424. doi:10.2307/1912791.

[12] Geweke J. Measurement of linear dependence and feedback between multiple time series. Journal of the American Statistical Association. 1982;77(378):304–313. doi:10.1080/01621459.1982.10477803.

[13] Barrett AB, Barnett L, Seth AK. Multivariate Granger causality and generalized variance. Physical Review E. 2010;81(4):041907.

[14] Mohsenipour AA. On the Distribution of Quadratic Expressions in Various Types of Random Vectors. The University of Western Ontario. Electronic Thesis and Dissertation Repository, 955.; 2012.

[15] Gutknecht AJ, Barnett L. Sampling distribution for single-regression Granger causality estimators; 2023. 4.

[16] Barnett L, Seth AK. Granger causality for state-space models. Physical Review E. 2015;91(4):040101.

[17] Barnett L, Seth AK. The MVGC multivariate Granger causality toolbox: A new approach to Granger-causal inference. Journal of Neuroscience Methods. 2014;223:50–68. doi:10.1016/J.JNEUMETH.2013.10.018.

[18] Barnett L, Seth AK. Dynamical independence: discovering emergent macroscopic processes in complex dynamical systems. Physical Review E. 2023;108(1):014304.

[19] Wilson GT. The factorization of matricial spectral densities. SIAM J Appl Math. 1972;23(4):420–426.

[20] Dhamala M, Rangarajan G, Ding M. Estimating Granger causality from Fourier and wavelet transforms of time series data. Phys Rev Lett. 2008;100:018701.

[21] Rozanov YA. Stationary Random Processes. San Francisco: Holden-Day; 1967.
