# Supplementary material for: Capturing the emergent dynamical structure in biophysical neural models
Source: PLoS Comput Biol. 2025 May 12;21(5):e1012572. doi: 10.1371/journal.pcbi.1012572 (PMC12068601; doi:10.1371/journal.pcbi.1012572)
Supplement: S2 Appendix — This appendix introduces state-space models as an alternative to VAR models for Granger causality estimation, highlighting their advantages in handling moving average components and providing computational methods for implementation. (PDF) [file pcbi.1012572.s002.pdf]

S2 Appendix: Linear state-space modelling

As described in S1 Appendix, dynamical dependence as a Granger causality may be estimated nonparametrically by estimation of the CPSD and spectral factorisation, or parametrically by vector autoregressive or linear state-space modelling. In this study, we use the linear state-space approach, along with the efficient spectral formula (Eq (12) in S1 Appendix) to calculate and minimise DD.

State-space models have a specific advantage over VAR models, with implications for Granger causality estimation: namely, that they are able to parsimoniously model[1] a moving average (MA) component in the data (they are in fact equivalent to the class of vector-autoregressive moving-average (VARMA) models [1]). This is of particular significance for modelling of neurophysiological data, since common technological and pre-processing factors such as observation noise, downsampling and digital filtering all induce an MA component in the data. Perhaps most importantly, even if a time series is generated by a pure finite-order VAR model, a *sub*-process of that process will generally have an MA component (and any recorded neural process is, of course, inevitably a sub-process of full brain dynamics). The sub-process issue has a particular impact on VAR GC estimation; even if the *full* process is pure, finite-order VAR, the *restricted* process will in general be VARMA. While this is somewhat mitigated by the single-regression VAR estimated mentioned previously, state-space modelling provides a more principled approach, yielding GC estimators of smaller bias and variance. See [2–5] for further discussions on these issues.

We consider observational data to be generated by a discrete-time, continuous-valued, wide-sense stationary stochastic vector process, $\boldsymbol{X} = [X^1, \ldots, X^N]^\top$ on $\mathbb{R}^N$. The linear state-space (SS) model framework assumes that the $\boldsymbol{X}_t$ are noisy observations of a latent (unobserved) state process $\boldsymbol{\xi}$ defined on $\mathbb{R}^r$, where the latent space dimension r may be larger or smaller than N. Specifically,

$$\boldsymbol{\xi}_{t+1} = A\boldsymbol{\xi}_t + \boldsymbol{\upsilon}_t \tag{1a}$$
$$\boldsymbol{X}_t = C\boldsymbol{\xi}_t + \boldsymbol{\nu}_t \tag{1b}$$

Here A is the stable[2] $r \times r$ state transition transition matrix, C the $N \times r$ observation matrix, while $\boldsymbol{\upsilon}$ is a white endogenous noise process, and $\boldsymbol{\nu}$ a white exogenous (observation) noise process; $\boldsymbol{\upsilon}$ and $\boldsymbol{\nu}$ may be contemporaneously correlated, with joint covariance matrix

$$\mathbb{E}\left[\begin{bmatrix}\boldsymbol{\upsilon}_t \\ \boldsymbol{\nu}_t\end{bmatrix}\begin{bmatrix}\boldsymbol{\upsilon}_t^\top & \boldsymbol{\nu}^\top\end{bmatrix}\right] = \begin{bmatrix} Q & S \\ S^\top & R\end{bmatrix} \tag{2}$$

The parameters of the model (1) are $\{A, C, Q, R, S\}$ (the covariance matrix $R = \mathbb{E}\big[\boldsymbol{\nu}_t\boldsymbol{\nu}_t^\top\big]$ must be positive-definite).

It may be shown that a state-space model in the form (1) may always be transformed to a convenient equivalent model in *innovations form* by introducing the new state variable $\boldsymbol{\zeta}_t = \mathbb{E}[\boldsymbol{\xi}_t \,|\, \boldsymbol{X}_{t-\tau}]$. The resulting ISS model takes the form

$$\boldsymbol{\zeta}_{t+1} = A\boldsymbol{\zeta}_t + K\boldsymbol{\varepsilon}_t \tag{3a}$$
$$\boldsymbol{X}_t = C\boldsymbol{\zeta}_t + \boldsymbol{\varepsilon}_t \tag{3b}$$

where K is the $r \times N$ *Kalman gain* matrix, and $\boldsymbol{\varepsilon}_t = \mathbb{E}[\boldsymbol{X}_t \,|\, \boldsymbol{X}_{t-\tau}]$ the *innovations* (white noise) process, with covariance matrix $\Sigma = \mathbb{E}\big[\boldsymbol{\varepsilon}_t\boldsymbol{\varepsilon}_t^\top\big]$. The parameters of the model (3) are then $\{A, C, K, \Sigma\}$, and our regularity conditions (specifically a

[1] That is, with lower model complexity.

[2] Our regularity conditions imply stability of A, which means that all its eigenvalues lie strictly within the unit circle in the complex plane.

minimum-phase requirement), require stability of $A - KC$. We may always transform a general SS model (1) to innovations form (3). We have

$$\Sigma = CPC^\top + R \tag{4a}$$

$$K = \big(APC^\top + S\big)\Sigma^{-1} \tag{4b}$$

where the $r \times r$ covariance matrix P is the unique stabilising solution of the Discrete-time Algebraic Riccati Equation (DARE)[3]:

$$P = APA^\top + Q - \big(APC^\top + S\big)\big(CPC^\top + R\big)^{-1}\big(CPA^\top + S^\top\big)\,. \tag{5}$$

There are efficient and stable computational algorithms available in major programming languages for solution of DAREs, and efficient algorithms too for estimation from time-series data of state-space models in innovations form[4] [refs].

The transfer function for the ISS model (3) is given by [2]:

$$H(z) = I + C(I - Az)^{-1}Kz\,, \tag{6}$$

from which the CPSD may be calculated according to Eq (8) in S1 Appendix.

Granger causality and dynamical dependence for state-space models

Since the innovations $\boldsymbol{\varepsilon}_t$ in an Innovations-form State Space (ISS) model are just the residual least-squares prediction errors $\mathbb{E}[\boldsymbol{X}_t \mid \boldsymbol{X}_{t-\tau}]$, the ISS form is well-suited to calculation of Granger causalities [2, 5]. Specifically, if we have an ISS model for a partitioned variable $\boldsymbol{U} = \big[\boldsymbol{X}^\top \boldsymbol{Y}^\top \boldsymbol{Z}^\top\big]^\top$, then by restricting the observation equation (3b), the restricted process $\boldsymbol{U}^{\mathsf{R}} = \big[\boldsymbol{Y}^\top \boldsymbol{Z}^\top\big]^\top$ is easily seen to follow an SS model, albeit no longer in SS form. We may then solve the appropriate DARE (5) to bring the restricted SS model into innovations form, and in particular to obtain Σ^{R} from (4b). We work through this procedure for dynamical dependence.

Given a state-space model in innovations form (3) and an orthogonal $n \times N$ linear coarse-graining matrix M, from (3b) the observation equation for $\boldsymbol{Y}_t = M\boldsymbol{X}_t$ is [2]:

$$\boldsymbol{Y}_t = M\boldsymbol{X}_t = MC\boldsymbol{\zeta}_t + M\boldsymbol{\varepsilon}_t\,. \tag{7}$$

This equation, along with the state-transition equation (3a), constitutes a restricted state-space model, though no longer in innovations form, for which $Q = K\Sigma K^\top$, $S = K\Sigma M^\top$, $R = M\Sigma M^\top$ and C is replaced by MC. We may then solve the corresponding DARE (5) for $P = P(M)$ to convert the restricted SS model to innovations form, which yields, in particular, the restricted residual error covariance matrix

$$\Sigma^{\mathsf{R}}(M) = MC \cdot P(M) \cdot C^\top M^\top + M\Sigma M^\top\,. \tag{8}$$

If the full residuals covariance matrix has been decorrelated/normalised to I, then this becomes simply

$$\Sigma^{\mathsf{R}}(M) = MC \cdot P(M) \cdot C^\top M^\top + I\,, \tag{9}$$

and the dynamical dependence is simply

$$F(\boldsymbol{X} \to M\boldsymbol{X}) = \log\big|\Sigma^{\mathsf{R}}(M)\big|\,. \tag{10}$$

Alternatively, we may calculate the CPSD $S(\omega)$ using the expression (6) for the ISS transfer function, and $F(\boldsymbol{X} \to M\boldsymbol{X})$ may then be calculated according to the spectral

[3] The DARE arises as the steady-state limit of the predictive Kalman filter for the SS model (1) [5].

[4] We recommend in particular the various "state-space-subspace" algorithms, which are non-iterative and yield quasi-log-likelihood estimates for the model parameters.

integral (Eq (12) in S1 Appendix). Anecdotally, we find that for neurophysiological time-series data, the latter approach is often more computationally efficient than the DARE route. It also facilitates a gradient descent approach to minimisation of dynamical dependence (S3 Appendix).

References

[1] Hannan EJ, Deistler M. The statistical theory of linear systems. SIAM; 2012.

[2] Barnett L, Seth AK. Granger causality for state-space models. Physical Review E. 2015;91(4):040101.

[3] Stokes PA, Purdon PL. A study of problems encountered in Granger causality analysis from a neuroscience perspective. Proceedings of the National Academy of Sciences. 2017;114(34):E7063–E7072. doi:10.1073/PNAS.1704663114.

[4] Barnett L, Barrett AB, Seth AK. Misunderstandings regarding the application of Granger causality in neuroscience. Proceedings of the National Academy of Sciences of the United States of America. 2018;115(29):E6676–E6677. doi:10.1073/PNAS.1714497115.

[5] Solo V. State-Space Analysis of Granger-Geweke Causality Measures with Application to fMRI. Neural Computation. 2016;28(5):914–949. doi:10.1162/NECO_A_00828.
