# Supplementary material for: Capturing the emergent dynamical structure in biophysical neural models
Source: PLoS Comput Biol. 2025 May 12;21(5):e1012572. doi: 10.1371/journal.pcbi.1012572 (PMC12068601; doi:10.1371/journal.pcbi.1012572)
Supplement: S3 Appendix — A gradient descent method for minimising dynamical dependence is presented, including a proxy function for efficiency and an adaptive step-size algorithm. Alternative optimisation strategies are also briefly discussed. (PDF) [file pcbi.1012572.s003.pdf]

S3 Appendix: Minimisation of dynamical dependence by gradient descent

A drawback with the DARE method (S2 Appendix) is that it is not clear how we might compute the gradient of the DD with respect to the Stiefel parametrisation by orthogonal matrices M, since the covariance matrix $P(M)$ in the expression Eq (9) of S2 Appendix for $\Sigma^{\mathsf{R}}(M)$ is defined only implicitly through solution of the restricted DARE. Gradient calculation, though, is indeed possible for the spectral form (Eq (12) in S1 Appendix), regardless of whether the CPSD $S(\omega)$ is acquired through parametric modelling (VAR or SS), or nonparametrically.

We note firstly that since the geometry of the Grassmannian manifold is non-Euclidean, calculation of the DD gradient under the Stiefel parametrisation by orthogonal matrices is not straightforward [1]. For macroscopic scale $n > 1$, furthermore, as remarked previously the Stiefel parametrisation is not one-to-one, with the implication that there will always be a zero-gradient submanifold in the DD cost surface over the Stiefel manifold[1]. For details of the Grassmannian gradient operator under the Stiefel parametrisation, see [1]; for calculation of the gradient of the spectral-form DD (Eq (12) in S1 Appendix), see [2] (APPENDIX D)[2]

A useful approach to speeding up the efficiency of state-space DD optimisation is pre-optimisation of the "proxy" DD:

$$F^*(\boldsymbol{X} \to M\boldsymbol{X}) = \sum_{k=0}^{r-1} \left\| M Q_k M^{\perp\top} \right\|^2 \tag{1}$$

where $M^{\perp}$ is a basis for the orthogonal complement of the hyperplane specified by M[3], $Q_k = CA^kK$, and $\|\cdot\|$ denotes the Frobenius matrix norm. Note that the Q_k depend only on the state-space model parameters, and may thus be precomputed. The proxy DD vanishes at exactly the same points on the Grassmannian as the actual state-space DD, and, as a polynomial in M, is computationally much cheaper to evaluate than both the DARE-derived and spectral-form DD. Its gradient may also be calculated explicitly. Empirically, the local minima of the proxy DD (1) appear in general to be found nearby on the Grassmannian to those of the actual DD. In [2] it is found that preliminary gradient descent using the proxy (1), followed by gradient descent of the spectral-form DD, initialised at the optimal hyperplane found by the proxy minimisation run, greatly improves optimisation efficiency.

A simple gradient descent algorithm with adaptive step size is deployed: an initial hyperplane is chosen uniformly at random on the Grassmannian, and the step size set to a specified initial value. At each iteration, a step is taken in the (downhill) direction of the gradient at the current hyperplane, using the current step size, and the DD (or proxy DD) evaluated there. If this DD is smaller than or equal to the current optimal DD, then the new hyperplane becomes the current one (the step is accepted), and the step size is increased by a fixed acceleration factor. Otherwise, the current hyperplane remains unchanged (the step is rejected), and the step size is decreased by a fixed deceleration factor. Optimisation terminates when the step size falls below a specified tolerance, or a maximum number of iterations is exceeded (optimisation times out).

[1]While there are indeed one-to-one parametrisations of the Grassmannian, they are inhomogeneous and not well-suited to implementation of gradient descent. In practice, however, the redundancy inherent in the Stiefel parametrisation does not appear to be a major impediment for gradient descent.

[2]We remark that it is also possible to calculate the appropriate *Hessian* (2nd-order differential operator) under the Stiefel parametrisation of the Grassmannian, and hence for the spectral-form DD, which in principle opens the way to 2nd-order gradient techniques such as Newton's method or conjugate gradient methods [1]. We found, though, that the computational costs of the Hessian calculation outweighed any gain in optimisation efficiency. We thus confine ourselves here to 1st-order methods.

[3]This may be calculated via a Singular Value Decomposition (SVD) of M.

Parameters for the algorithm are the initial step size, the acceleration and deceleration factors, the step size termination tolerance, and the maximum number of iterations. See [2] for full details and discussion of alternative optimisation techniques. An open-source MATLABimplementation of state-space dynamical dependence calculation and minimisation techniques as described here may be obtained from https://github.com/lcbarnett/ssdi.

References

[1] Edelman A, Tom T, Arias TA, Smith ST. The Geometry Of Algorithms Wwith Orthogonality Constraints. Society for Industrial and Applied Mathematics. 1998;20(2):303–353.

[2] Barnett L, Seth AK. Dynamical independence: discovering emergent macroscopic processes in complex dynamical systems. Physical Review E. 2023;108(1):014304.
