# Supplementary material for: Capturing the emergent dynamical structure in biophysical neural models
Source: PLoS Comput Biol. 2025 May 12;21(5):e1012572. doi: 10.1371/journal.pcbi.1012572 (PMC12068601; doi:10.1371/journal.pcbi.1012572)
Supplement: S4 Appendix — The evolution equation governing whole-brain models is outlined, incorporating structural connectivity and time delays. The Stefanescu-Jirsa 3D neural mass model is introduced with its mathematical formulation and role in macroscopic dynamics. (PDF) [file pcbi.1012572.s004.pdf]

S4 Appendix: Global brain network dynamics

Globally, the brain network dynamics are governed by an *evolution equation* which takes in the activity of the entire system at time t and outputs the vector-valued state of the biophysical neural model at time $t+1$. Consider a single node x_i within this framework. The dynamical process at node x_i over time is described by the following equation:

$$\frac{dx_i(t)}{dt} = N(x_i(t)) + G\sum_j SC_{ij}x_j(t - d_{ij}^{glob}) + I_i(t) + v_i(t) \tag{1}$$

Eq 1 is the same evolution equation used for whole-brain network simulations [1]. Table A provides definitions for the terms in Eq 1 as such:

Table A: **Evolution equation parameters**

Parameter	Description
$\frac{dx_i(t)}{dt}$	The differential operator mapping system of equations into its derivative i.e. its next time step.
N	State operator for each neural mass model, determining the internal dynamics of node.
$x_i(t)$	Vector of state variables for each region (e.g., mean field, or firing rate).
G	Global connectivity scaling factor, modulating the influence of global connections on the node's activity.
SC_{ij}	Structural connectivity matrix (global coupling in the WBM, at level $\nu = 2$
d_{ij}^{glob}	Time delay between globally coupled nodes, representing the propagation time of signals across the brain network.
$I_i(t)$	External input to the node, representing external stimuli or perturbations affecting the system.
$v_i(t)$	Noise term, accounting for stochastic fluctuations in the system, capturing the inherent variability in neural activity.

Two crucial elements in this model are essential. First, temporal delays are included on global connections, providing a more realistic understanding of how brain activity propagates at specific nodes [2–4]. These delays are particularly critical at the WBM level, where interactions between localised brain areas unfold over time on the mesoscopic level, leading to the large-scale, macroscopic, spatiotemporal dynamics observed in the whole brain. Second, a veridical structural connectivity matrix implements the large-scale network. This matrix is usually obtained from magnetic resonance imaging (MRI) scans.

In summary, WBMs are versatile, accommodating both local interactions within cortical areas and global interactions across the entire brain. This allows researchers to model how neural dynamics evolve both locally and globally, providing insights into the mesoscopic processes that give rise to patterns of macroscopic activity.

Neural mass model

The Stefanescu-Jirsa 3D (SJ3D) model is derived from a mean-field approximation of coupled Hindmarsh-Rose (HR) neuronal models. HR neurons are excitable neuronal models that are capable of exhibiting neuronal bursting oscillations, endowing the subsequent SJ3D model with capability of displaying qualitatively complex dynamics

such as synchronisation, metastability, multi-clustering, spike-burst behaviour, oscillatory death and spiking limit-cycle oscillations [1, 5, 6].

The HR model consists of three coupled differential equations:

$$\frac{dx}{dt} = y - ax^3 + bx^2 - z + I \tag{2a}$$

$$\frac{dy}{dt} = c - dx^2 - y \tag{2b}$$

$$\frac{dz}{dt} = r\big(s(x - x_0) - z\big) \tag{2c}$$

Here, x and y are state-variables that evolve on a faster time-scale, dictating the spike-burst activity, and z is a state-variable representing the slower time-scale changes usually representative of the oscillatory activity. I is an external input parameter and is responsibly for altering the system dynamics from fixed-point dynamics ($I < 1.3$), spike-bursting behaviour ($I > 1.3$). Which additionally, can be expressed in a chaotic regime or an oscillatory regime.

Given that we want to describe two neural masses (populations) within one neural mass model, we can therefore adapt the HR model for population dynamics as such:

$$\dot{x}_{i1} = y_{i1} - ax_{i1}^3 + bx_{i1}^2 - z_{i1} + \Bigg(K_{11}(X_2 - x_{i1}) - K_{12}(X_2 - x_{i1})\Bigg) + I_{i1} \tag{3a}$$

$$\dot{y}_{i1} = c - dx_{i1}^2 - y_{i1} \tag{3b}$$

$$\dot{z}_{i1} = r\big(s(x_{i1} - x_{i0}) - z_{i1}\big) \tag{3c}$$

$$\dot{x}_{j2} = y_{j2} - ax_{j2}^3 + bx_{j2}^2 - z_{j2} + K_{21}(X_1 - x_{j2}) + I_{j2} \tag{3d}$$

$$\dot{y}_{j2} = c - dx_{j2}^2 - y_{j2} \tag{3e}$$

$$\dot{z}_{j2} = r\big(s(x_{j2} - x_{j0}) - z_{j2}\big) \tag{3f}$$

$$\tag{3g}$$

Above, the mesoscopic coupling between excitatory and inhibitory neural masses is indicated by the K_{nm} parameters and is given in Table B. Given mode decomposition methods as presented in Assisi *et al.* [7], the following reduced equations are derived by the original authors [6]:

$$\dot{\xi}_i = \eta_i - a_i\xi_i^3 + b_i\xi_i^2 + K_{11}\left[\sum_{k=1}^{3} A_{ik}\xi_k\right] - K_{12}\left[\sum_{k=1}^{3} B_{ik}\alpha_k - \xi_i\right] + IE_i + \left[\sum_{k=1}^{3}\Gamma(\xi_{kq}, \xi_{kr}, u_{qr})\right] + \left[\sum_{k=1}^{3} W_\zeta \cdot \xi_{kr}\right] \tag{4a}$$

$$\dot{\eta}_i = c_i - d_i\xi_i^2 - \eta_i \tag{4b}$$

$$\dot{\tau}_i = rs\xi_i - r\tau_i - m_i \tag{4c}$$

$$\dot{\alpha}_i = \beta_i - e_i\alpha_i^3 + f_i\alpha_i^2 - \gamma_i + K_{21}\left[\sum_{k=1}^{3} C_{ik}\xi_k - \alpha_i\right] + II_i + \left[\sum_{k=1}^{3}\Gamma(\xi_{kq}, \xi_{kr}, u_{qr})\right] + \left[\sum_{k=1}^{3} W_\zeta \cdot \xi_{kr}\right] \tag{4d}$$

$$\dot{\beta}_i = h_i - p_i\alpha_i^2 - \beta_i \tag{4e}$$

$$\dot{\gamma}_i = rs\alpha_i - r\gamma_i - n_i \tag{4f}$$

Now, the connectivity on the macroscopic scale of brain regions is given introduced by the W_ζ parameter. The full description of the parameters adapted from [8] is given in Table B. For the interested reader, the comparison between the full model (Eq 3 and the reduced equations (Eq 4) is performed in the original articles [5, 6].

Table B also includes the range of global coupling and dynamical noise for the bivariate parameter sweep. Moreover, $\mathbf{V}_{\nu=2}$. Here, following the original notation of Sanz-Leon [1], $\mathbf{V}_{\nu=2}$ corresponds to the inter-regional connectivity between brain regions or nodes, and $\mathbf{V}_{\nu=1}$ corresponds to the intra-regional connectivity between excitatory and inhibitory populations with the individual NMMs.

Table B: **Local Stefanescu-Jirsa 3D and Global Network Parameters**

Stefanescu-Jirsa 3D neural mass model		
Parameter	*Value*	*Description*
Hindmarsh-Rose neuron parameters		
r	0.006	Controls the speed of the variation of the slow variables z and u
s	4	Adaptation variable
x_0	-1.6	Membrane resting potential
a	1	Models the behaviour of the fast ion channels
b	3	Models the behaviour of the fast ion channels
c	1	Models the behaviour of the fast ion channels
d	5	Models the behaviour of the fast ion channels
Local, intrinsic neural mass network parameters		
K_{11}	0.5	Excitatory to excitatory coupling strength (i.e. recurrent connections)
K_{12}	0.15	Inhibitory to excitatory coupling strength
K_{21}	0.15	Excitatory to inhibitory coupling strength
σ	0.3	Standard deviation of Gaussian distribution of membrane excitability for both, excitatory and inhibitory masses
μ	2.2	Mean of of Gaussian distribution of membrane excitability for both, excitatory and inhibitory masses
Property	Value	
d (dimension)	18	
m (masses)	2	
o (modes)	3	
n (state variables)	3 variables describing each neural mass (ξ,η,τ,α,β,γ)	
$\mathbf{V}_{\nu=2}$	$\begin{array}{ccccccc} & \xi^j & \eta^j & \tau^j & \alpha^j & \beta^j & \gamma^j \\ \xi^i & 1 & 0 & 0 & 0 & 0 & 0 \\ \eta^i & 0 & 0 & 0 & 0 & 0 & 0 \\ \tau^i & 0 & 0 & 0 & 0 & 0 & 0 \\ \alpha^i & 1 & 0 & 0 & 0 & 0 & 0 \\ \beta^i & 0 & 0 & 0 & 0 & 0 & 0 \\ \gamma^i & 0 & 0 & 0 & 0 & 0 & 0 \end{array}$	
$\mathbf{V}_{\nu=1}$	$\begin{array}{ccccccc} & \xi^j & \eta^j & \tau^j & \alpha^j & \beta^j & \gamma^j \\ \xi^i & 1 & 0 & 0 & 1 & 0 & 0 \\ \eta^i & 0 & 0 & 0 & 0 & 0 & 0 \\ \tau^i & 0 & 0 & 0 & 0 & 0 & 0 \\ \alpha^i & 1 & 0 & 0 & 1 & 0 & 0 \\ \beta^i & 0 & 0 & 0 & 0 & 0 & 0 \\ \gamma^i & 0 & 0 & 0 & 0 & 0 & 0 \end{array}$	
Classification	Phenomenological Model	Voltage-based
Parameter sweep variables		
Coupling (G)	(0.01, 0.31)	Range on log-scale
Noise (η)	(0.001, 0.1)	Range on log-scale

References

[1] Sanz-Leon P, Knock SA, Spiegler A, Jirsa VK. Mathematical framework for large-scale brain network modeling in The Virtual Brain. NeuroImage. 2015;111:385–430. doi:10.1016/J.NEUROIMAGE.2015.01.002.

[2] Ghosh A, Rho Y, McIntosh AR, Kötter R, Jirsa VK. Noise during Rest Enables the Exploration of the Brain's Dynamic Repertoire. PLOS Computational Biology. 2008;4(10):e1000196. doi:10.1371/JOURNAL.PCBI.1000196.

[3] Deco G, Jirsa VK, McIntosh AR. Resting brains never rest: computational insights into potential cognitive architectures. Trends in neurosciences. 2013;36(5):268–274.

[4] Honey CJ, Kötter R, Breakspear M, Sporns O. Network structure of cerebral cortex shapes functional connectivity on multiple time scales. Proceedings of the National Academy of Sciences. 2007;104(24):10240–10245.

[5] Stefanescu R, Jirsa V. A low dimensional description of globally coupled heterogeneous neural networks of excitatory and inhibitory neurons. PLoS Comput Biol. 2008;4(11):e1000219–e1000219. doi:10.1371/journal.pcbi.1000219.

[6] Stefanescu R, Jirsa V. Reduced representations of heterogeneous mixed neural networks with synaptic coupling. Physical review E, Statistical, nonlinear, and soft matter physics. 2011;83(2 Pt 2):026204–026204. doi:10.1103/physreve.83.026204.

[7] Assisi C, Jirsa V, Kelso J. Synchrony and clustering in heterogeneous networks with global coupling and parameter dispersion. Physical Review Letters. 2005;94(1):018106–018106. doi:10.1103/physrevlett.94.018106.

[8] Ritter P, Schirner M, McIntosh AR, Jirsa VK. The Virtual Brain Integrates Computational Modeling and Multimodal Neuroimaging. Brain Connectivity. 2013;3(2):121. doi:10.1089/BRAIN.2012.0120.

[9] Wong YC. Differential geometry of Grassmann manifolds. Proceedings of the National Academy of Sciences. 1967;57(3):589–594.
