# Supplementary material for: Capturing the emergent dynamical structure in biophysical neural models
Source: PLoS Comput Biol. 2025 May 12;21(5):e1012572. doi: 10.1371/journal.pcbi.1012572 (PMC12068601; doi:10.1371/journal.pcbi.1012572)
Supplement: S5 Appendix — Principal angles are used to quantify similarity between subspaces and identify emergent macroscopic structures. The contribution of individual and grouped microlevel nodes to macroscopic dynamics is analysed, with implications for understanding emergent structures in brain activity. (PDF) [file pcbi.1012572.s005.pdf]

S5 Appendix: Principal angles & single-node contribution to macroscopic dynamics

Principal angles between subspaces, specifically macroscopic variables of dimension n, can be used to define invariant metrics on the corresponding Grassmannian manifold [1]. Formally, given two subspaces in $\mathbb{R}^n$ a metric on the Grassmannian can be defined as $\sqrt{\theta_1^2 + \theta_2^2 + \cdots + \theta_n^2}$, where each $\theta_i \in (0, \pi/2)$ represents a principal angle corresponding to the coordinate axes of an n-dimensional macroscopic variable. Hereafter, these macroscopic variables are denoted as n-macros.

To quantify the similarity between two n-macros of the same dimension, obtained from different optimisation runs with random initial restarts, we define a metric that is normalised within the interval $[0, 1]$. Here, 0 represents complete coplanarity (generalised colinearity), while 1 indicates orthogonality between the n-macros. Specifically, for a n-macro, the similarity metric is given by $\left(\frac{\pi}{2}\right)^{-1} \sqrt{\theta_1^2 + \theta_2^2 + \theta_3^2}$, where perfect coplanarity (identical n-macros) results in a metric value of 0.

This metric forms the foundation for identifying the *emergent dynamical structure* associated with higher-order interactions across spatial scales. By exploiting the optimisation landscape's structure—specifically, the geometry of the Stiefel manifold—we determine whether local minima dominate the energy landscape. Since these local minima correspond to n-macros, they provide a proxy for assessing the dynamical dominance, and hence, the emergent dynamical structure at higher-order scales.

Similarly, using principal angles, we can evaluate the extent to which the dynamics are *localised* within the original microscopic state-space.

Localisation of n-macros to microlevel nodes: a single-node approach

Given the identified emergent n-macros and their corresponding DD values, a natural question arises: *Which microlevel nodes contribute to the dynamics of the n-macro, and to what extent*? This consideration enables the projection of n-macros back onto the microscopic base, or causal graph, to determine where the macroscopic dynamics are localised within the original microscopic state space. This provides a specific perspective on the *emergent dynamical structure* by the degree of distinct localisation of the macroscopic processes on the constituents of the microscopic process.

Similarly to measuring the principal angles between subspaces (n-macros) on the same spatial scale, we can measure the principal angles between the coordinate axes of the N-dimensional microscopic base and a candidate n-macro. This generalisation of principal angles applies to any two subspaces of arbitrary dimensions. The degree of single-node contribution is now represented by $1-$ the metric distance: $1 - \left(\frac{\pi}{2}\right)^{-1} \sqrt{\theta_1^2 + \theta_2^2 + \cdots + \theta_n^2}$. In this case, a value closer to 1 indicates a higher degree of single node contribution to the n-macro.

To formally define what it means for a microscopic node to contribute to the n-macro, consider a system of three variables $\{X^1, X^2, X^3\}$ undergoing a linear transformation induced by an emergent 2-macro at time t. This can be expressed as the matrix multiplication:

$$\boldsymbol{Y} = M\boldsymbol{X} = \begin{bmatrix} M_{11} & M_{12} & M_{13} \\ M_{21} & M_{22} & M_{23} \end{bmatrix} \begin{bmatrix} X_t^1 \\ X_t^2 \\ X_t^3 \end{bmatrix} = \begin{bmatrix} M_{11}X_t^1 + M_{12}X_t^2 + M_{13}X_t^3 \\ M_{21}X_t^1 + M_{22}X_t^2 + M_{23}X_t^3 \end{bmatrix} \quad (1)$$

Here, N and n represent the dimensions of the microscopic base and the n-macro, respectively. Each element M_{nN} parameterises the 2-macro and corresponds to the element in the Stiefel manifold represented by the orthonormal matrix M, determining

the contribution of each microscopic node X_t^N to the macroscopic process. For instance, if $M_{11} = M_{21} = 0$, the macroscopic dynamics governed by the 2-macro are entirely driven by X^2 and X^3. In the context of a brain network, where X^2 and X^3 represent two regions in a three-region network, this indicates that these two regions contribute to the emergent 2-macro, while the X^1 region does not.

Collectively, the 2-macro and the single node X^1 encapsulate the the localisation of the macroscopic variable, and therefore a perspective of the emergent dynamical structure of this simplified system. This provides a dimensionally reduced description of the dynamics without resorting to the full higher-dimensional microscopic dynamics. The free parameters M_{nk} define the principal angle between any n-macro and the microscopic base $\boldsymbol{X}$.

Localisation of n-macros to microlevel nodes: a grouped-node approach

In higher-dimensional systems, the relationship between the coordinate axes of n-macros and those of the microscopic system becomes more complex, as the specificity of which nodes contribute to the n-macro is harder to determine. Formally, this reflects the fact that the set of subspace angles (principal angles) between an n-macro and the coordinate axes of the microlevel constituents does not uniquely identify the n-macro. In higher dimensions, distinct subspaces (n-macros) can share the same set of angles with the coordinate axes, and can sometimes lead to ambiguity in pinpointing the contributing microlevel nodes [2].

Given the high dimensionality of neurophysiological recordings or region-based whole-brain models—often encompassing between 32 and 256 variables—subspace angles between each individual node of the microscopic process and n-macros may not uniquely determine the localisation of the emergent dynamical structure in the microscopic state-space. However, despite these challenges, assessing the contribution of individual nodes to n-macros remains computationally tractable and highly informative, especially when a researcher seeks a fine-grained understanding of the microlevel localisation of the n-macros.

As an alternative, we could impose an additional constraint by measuring the subspace angle between each emergent n-macro and all n-combinations of microlevel variables. Formally, this is defined as the subspace angle between the emergent n-macro and all n-subsets of the original N-dimensional microlevel state-space, thus there are $\binom{N}{n}$ such combinations. Unlike angles with coordinate axes, these $\binom{N}{n}$ subspace angles do uniquely specify the subspace—though at the cost of a combinatorial explosion. While this approach offers less specificity than measuring the subspace angle between each node and n-macro, it serves as a proxy for partitioning the system into distinct emergent cores.

The n-dimensional subset that is optimally implicated in driving the macroscopic dynamics is identified as the n-subset with the minimal principal angle from the n-macro. Though illustrated here, this alternative approach is not utilised in the present study because of its focus on hard-partitioning the biophysical network.

References

[1] Wong YC. Differential geometry of Grassmann manifolds. Proceedings of the National Academy of Sciences. 1967;57(3):589–594.

[2] Barnett L, Seth AK. Dynamical independence: discovering emergent macroscopic processes in complex dynamical systems. Physical Review E. 2023;108(1):014304.
