# Supplementary material for: Capturing the emergent dynamical structure in biophysical neural models
Source: PLoS Comput Biol. 2025 May 12;21(5):e1012572. doi: 10.1371/journal.pcbi.1012572 (PMC12068601; doi:10.1371/journal.pcbi.1012572)
Supplement: S6 Appendix — A clear summary of technical terms used within the paper. (PDF) [file pcbi.1012572.s006.pdf]

S6 Appendix: Glossary

Glossary

Coarse-Graining: A technique that reduces the dimensionality of a system by grouping together similar states or variables into a single, larger-scale description.
Biophysical Model: A computational model that incorporates biological realistic details, capturing the physical and biological mechanisms underlying a system, such as neural dynamics.
Dynamical Independence (DI): A measure of how independent the dynamics constrained within a macroscopic variable are from the underlying microlevel constituent units.
Distributed / Local: Refers to whether a macroscopic process is spread across many variables (distributed) or confined to a few variables (local).
Emergence: The phenomenon where higher scale patterns, processes, or properties arise from interactions of lower scale constituent units.
Emergent Dynamical Structure: The collection of macroscopic *patterns* of activity across spatial scales that emerge from the interactions between lower scale constituent units.
Evolution Equation: A mathematical equation that describes how a system's state changes over time, often used in neural models to represent dynamic processes.
Integration: The tendency of different parts of a system to work together to form a cohesive whole, often through coupling between units.
Higher-order Scales: Spatially macroscopic descriptions of a system that captures the emergent dynamics arising from interactions at the lower scales between more than 2 variables.
Macroscopic Variable: A higher-scale variable that describes the collective behaviour of many microscopic variables.
Microscopic Variable: A small-scale variable that represents the state or behavior of a component in a system.
Neural Mass Model (NMM): A simplified mathematical model that represents the collective dynamics of neural populations, often used to describe brain activity.
Parameter Sweep: The process of systematically varying parameters in a model to observe their effects on the system's behaviour.
Partition: A division of the state-space into distinct, non-overlapping subsets.
Segregation: The tendency of parts of a system to behave independently, reducing the interactions and coupling between them.
State-Space: The set of all possible states that a system can occupy and the dynamics between them.
Stochastic Process: A process that involves randomness, where the next state is not fully determined by a set of previous states in it's past.
Surjective: A function that maps every element in the target set (output) to at one or more elements in the domain (input).
